# Whole-transcriptome profiling across different developmental stages of *Aedes albopictus* (Diptera: Culicidae) provides insights into chitin-related non-coding RNA and competing endogenous RNA networks

**DOI:** 10.1186/s13071-022-05648-2

**Published:** 2023-01-26

**Authors:** Wenjuan Liu, Sha An, Peng Cheng, Kexin Zhang, Maoqing Gong, Zhong Zhang, Ruiling Zhang

**Affiliations:** 1 School of Clinical and Basic Medical Sciences, Shandong First Medical University (Shandong Academy of Medical Sciences), Jinan, 250117 China; 2 School of Laboratory Animal (Shandong Laboratory Animal Center), Shandong First Medical University (Shandong Academy of Medical Sciences), Jinan, 250117 China; 3 Shandong Institute of Parasitic Diseases, Shandong First Medical University (Shandong Academy of Medical Sciences), Jining, 272033 China

**Keywords:** ceRNA, ncRNA, Chitin, Mosquito

## Abstract

**Background:**

The Asian tiger mosquito, *Aedes albopictus*, is one of the most invasive species and a vector of numerous arboviruses. The deleterious effects of long-term and inappropriate use of chemical pesticides have stimulated the exploration of new, environmentally friendly control strategies. Non-coding RNAs (ncRNAs) have been proven to participate in almost all biological processes of insects.

**Methods:**

In this study, circular RNAs (circRNAs) and microRNAs (miRNAs) covering five developmental stages [egg, early larvae, late larvae, pupae, adult (female and male)] of *A. albopictus* were obtained using whole-transcriptome sequencing technology. Combined with long non-coding RNAs (lncRNAs) from previous research, circRNA/lncRNA‒miRNA‒mitochondrial RNA (mRNA) networks were constructed.

**Results:**

A total of 1434 circRNAs and 208 miRNAs were identified. More differentially expressed circRNAs (DE circRNAs) and miRNAs (DE miRNAs) were found in the egg versus early larvae comparison group. Functional enrichment analysis demonstrated that most of the circRNA/lncRNA‒miRNA‒mRNA networks were involved in chitin metabolism. Hub genes of each circRNA/lncRNA‒miRNA‒mRNA network were screened out, which can be used as novel targets to disturb the molting process of *A. albopictus*.

**Conclusions:**

Regulatory relationships obtained from competing endogenous RNA (ceRNA) networks provide more information to manipulate the metamorphosis process and are helpful for developing effective and sustainable methods to control mosquitoes.

**Graphical Abstract:**

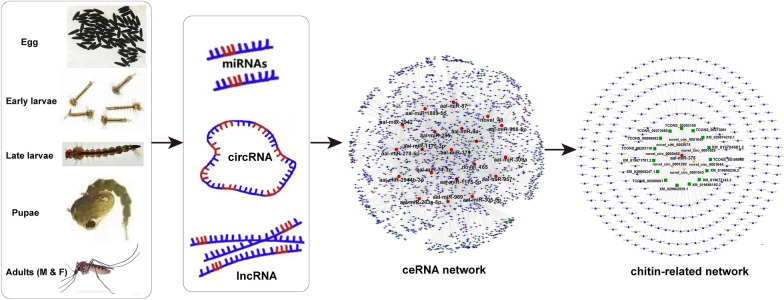

**Supplementary Information:**

The online version contains supplementary material available at 10.1186/s13071-022-05648-2.

## Background

*Aedes albopictus* originated in Southeast Asia and has now spread from its area of origin to more than 70 countries and regions around the world [1]. As one of the important vectors, *A. albopictus* can transmit dengue virus, Zika virus, chikungunya virus, yellow fever virus, and some other arboviruses, posing a huge threat to public health [2]. Despite decades of control efforts, mosquito-borne diseases are a major threat to public health in large regions of the world. Dengue virus causes an estimated 400 million infections annually, with 3.6 billion people living in areas at risk for epidemic transmission [2]. There is still no effective vaccine or drug for the diseases caused by viruses transmitted via *A. albopictus* [3]. Mosquito population control is the most effective strategy for preventing mosquito-borne diseases. Conventional control methods depend primarily on insecticides, which have been proven to be unsustainable solutions for a variety of reasons, such as rapidly increasing insecticide resistance [4] and negative impacts on the ecological environment [5]. Therefore, the development of a novel control strategy with proven epidemiological impact is challenging.

Non-coding RNAs (ncRNAs) are functional RNA molecules that cannot be translated into proteins [6]. MicroRNAs (miRNAs), long non-coding RNAs (lncRNAs), and circular RNAs (circRNAs) are major families of ncRNAs. With the development of sequencing and experimental technology in recent years, a large number of studies have demonstrated that ncRNAs play important roles in the developmental regulation of insects [7–9]. miRNAs are small RNA molecules with 21–23 nucleotides that regulate gene expression post-transcriptionally, mainly by binding the mRNA target. lncRNAs are a heterogeneous class of transcripts that are more than 200 nucleotides in length and lack the potential for protein coding [10–12]. circRNA is a newly discovered kind of endogenous ncRNA with covalently closed loop structures formed by back-splicing, which exhibits high abundance, stability, species conservation, and tissue/stage specificity [13–15].

Accumulating evidence indicates that ncRNAs exert their biological activity in the reproduction, embryogenesis, molting, development, and insecticide resistance of Arthropoda [16–19]. These ncRNA-mediated interactions are often interconnected, constructing complex regulatory RNA networks [12, 19, 20]. This complicated regulatory network is described as competing endogenous RNAs (ceRNAs), which play important regulatory roles in post-transcriptional gene expression [21, 22]. The ceRNA network has been described as an intricate interplay among diverse RNAs [23]. In addition to mRNAs, lncRNAs, circRNAs, and other RNAs that share common miRNA response elements (MREs) can competitively bind with miRNAs, acting as an RNA sponge to block and inhibit miRNAs from binding to their target sites [23]. These ceRNA modulatory mechanisms have been found to participate in many biological processes. For example, ceRNA (circRNA–miRNA–mRNA) networks have been shown to play a comprehensive role in the growth, development, and metabolism in the midgut of the *Apis cerana cerana* workers [7]. lincRNA_Tc13743.2-miR-133-5p-TcGSTm02 is involved in mediating cyflumetofen resistance in *Tetranychus cinnabarinus* [24]. lnc-GSTu1_AS-miR-8525-5p-GSTu1 were also found to regulate the expression of detoxifying enzymes (glutathione S-transferase, GST) to mediate the resistance of *Plutella xylostella* to chlorantraniliprole [25].

A deep understanding of the expression profile and regulatory relationships of ncRNAs during the development of *A. albopictus* will strongly improve our knowledge of the molecular mechanisms underlying the holometabolous development of the mosquito. Because of the significant heterogeneity of ncRNAs, we performed a comparative analysis of miRNAs and circRNAs covering five developmental time points during the life-cycle of *A. albopictus*. Combining the lncRNA data in previous studies [26], ceRNA networks (circRNA/lncRNA‒miRNA‒mRNA) were established for each stage. Meanwhile, the hub genes of each ceRNA network were screened out, and a subnetwork of pivotal hub genes was also constructed. The results of this study will offer a novel perspective for investigating post-transcriptional regulatory mechanisms of ncRNAs underlying developmental processes, promoting the discovery of innovative control strategies, such as the use of hub genes as developmental time-specific tuners to regulate chitin metabolism and ultimately control of *A. albopictus*.

## Methods

### Sample preparation

The *A. albopictus* colony used in this study was collected in Shandong Province (China) and reared in the laboratory under conditions of 27 ± 1 °C and 65% relative humidity (RH) with a daily photoperiod of 14:10 h (light/dark). Eggs were collected within 24 h after damp collection filter paper was placed into an insect cage, and were pooled to represent the embryonic stage. Larvae were reared in dechlorinated tap water in plastic containers and fed a slurry that was a mixture of pork liver powder (homemade), yeast, and distilled water. Larvae samples were divided into early (first–second-instar) and late (third–fourth-instar) larval stages. Pupae samples were mixed pools of various stages. Individual pupae were placed in plastic tubes equipped with dechlorinated tap water and covered with absorbent cotton until adults emerged. Then, male and female adults were collected separately. Three duplicate samples for each stage were prepared. All samples were flash frozen in liquid nitrogen immediately following the collection and then stored at −80 °C until RNA isolation.

### RNA extraction, library construction, and sequencing

Total RNA was extracted from *A. albopictus* samples (egg, early larvae, late larvae, pupae, adults [female and male]) using TRIzol reagent (Invitrogen, USA). RNA purity was checked using a NanoPhotometer^®^ spectrophotometer (Implen, Germany), and the RNA concentration was measured using a Qubit^®^ RNA Assay Kit in a Qubit^®^ 2.0 Fluorometer (Life Technologies, USA). RNA integrity was assessed using the RNA Nano 6000 Assay Kit of the Agilent Bioanalyzer 2100 system (Agilent Technologies, USA). An Epicenter Ribo-Zero™ rRNA Removal Kit (Epicenter, USA) was used to remove ribosomal RNA, and the rRNA-free residue was cleaned up by ethanol precipitation.

circRNA library preparation was carried out using the NEBNext^®^ Ultra™ Directional RNA Library Prep Kit for Illumina R (NEB, USA) following the manufacturer’s recommendations. RNA sequencing (RNA-Seq) was performed by Novogene (Beijing, China) using the Illumina HiSeq 2000 platform, and 150-base-pair (bp) paired-end reads were generated. NEBNext^®^Multiplex Small RNA Library Prep Set for Illumina^®^ (NEB, USA) was used for miRNA sequencing library construction according to the manufacturer’s protocol. Single-end reads (50 bp) were generated using the Illumina HiSeq 2500 platform.

Clean reads were obtained by removing the adaptor reads and low-quality tags (containing ploy-N, 5′ adapter contaminants, without 3′ adapter or the insert tag, containing ploy A or T or G or C and low-quality reads) from the raw reads using Trimmomatic v0.38 [27]. All subsequent analyses were performed based on the clean reads. The Q30 and guanine-cytosine (GC) content of the samples were calculated. The miRNAs were mapped to the *A. albopictus* genome (genome version: AalbF2, assembly: GCA_006496715.1, NCBI) [28] by Bowtie 2 v 2.4.4 [29]. circRNAs were mapped to the reference genome (genome version: AalbF2) using HISAT2 v2.1.0 [30].

### Identification of circRNA and miRNA

Both find_circ [14] and CIRI2 [31] were used to detect and identify circRNAs. Known miRNAs were identified according to miRBase 22.1 [32]. The prediction of novel miRNA was performed by miREvo [33] and mirdeep2 [34] based on the secondary structure (hairpin structure, Dicer cleavage sites, and minimum free energy) of miRNA. Then, miFam.dat (http://www.mirbase.org/ftp.shtml) and Rfam (http://rfam.sanger.ac.uk/search/) were used to search for families of known miRNAs and Rfam families of novel miRNAs, respectively. Target gene prediction of miRNA was performed by miRanda [35].

### Identification of differentially expressed circRNAs (DE circRNAs) and miRNAs (DE miRNAs)

The expression levels of circRNA and miRNA were estimated by transcripts per million (TPM) according to the criteria of Zhou et al. [36]. Differential expression analysis was performed using DESeq R v3.1.3 [37]. The *P*-value was adjusted using the Benjamini‒Hochberg method [38]. The threshold for significantly differential expression was set as |log2 (fold change)|> 1.0 and adjusted *P*-value < 0.05. Venn diagrams were drawn to depict the circRNA and miRNA expression profiles at different developmental time points.

### Establishment of ceRNA networks

Both miRanda and RNAhybrid software [39] were used to predict the relationships among DE miRNAs, circRNAs, and lncRNAs. Only relationships that were identified simultaneously by these two methods were used for subsequent analysis. A ceRNA network was constructed with potential target mRNAs to the miRNAs and potential target circRNAs/lncRNAs to the miRNAs and corresponding mRNAs. The nodes in the network represent the genes, and the nodes are connected if the corresponding genes are significantly co-expressed [40]. Gene connectivity was represented by edge weight, defined as the sum of the weights across all edges of a node in the gene co-expression network analyzed. Hub genes were identified based on the connectivity degree of genes in the network with the Cytohubba plugin [41] in Cytoscape v3.8.2 [42].

### Functional enrichment analysis

Gene Ontology (GO) and Kyoto Encyclopedia of Genes and Genomes (KEGG) enrichment analyses of the target gene candidates of DE circRNAs, DE miRNAs, and DE mRNAs in the lncRNA/circRNA–miRNA–mRNA network were implemented using the GOseq R v3.3.2 package [43] and KOBAS v3.0 software [44], respectively. A *P*-value < 0.05 was considered significantly enriched.

### Reverse transcription real-time quantitative polymerase chain reaction (qRT‒PCR) analysis

To verify the accuracy of the results obtained from high-throughput sequencing, four ceRNA co-expression networks were randomly selected. The expression profiles of ncRNAs and mRNAs in these selected ceRNA networks were confirmed using qRT‒PCR, which used the same RNA samples as Illumina sequencing.

Total RNA was isolated using the TRIzol reagent (Vazyme, China) according to the manufacturer’s protocol. The miRNA reverse transcription was performed using a miRNA first-strand cDNA synthesis kit (by stem-loop) (Vazyme, China). The lncRNA and mRNA template was reversely transcribed into complementary DNA (cDNA) using HiScript III RT SuperMix for qPCR (+ gDNA wiper) (Vazyme, China). Then, qPCR of miRNA was performed with miRNA Universal SYBR qPCR Master Mix (Vazyme, China); qPCR of circRNA, mRNA, and lncRNA was performed using ChamQ Universal SYBR qPCR Master Mix (Vazyme, China). All qRT-PCR runs were carried out on an ABI 7500 qRT-PCR platform (Thermo Fisher Scientific, USA). The expression levels of the selected miRNAs, circRNAs, lncRNAs, and mRNAs were normalized against the β-actin. All experiments were performed with three biological and technical replicates, respectively. The 2^−ΔΔCT^ method was used to estimate the relative expression of each lncRNA [45].

## Results

### Identification of circRNAs and miRNAs

Altogether, 18 libraries covering five developmental time points (with three biological replicates for each time point) were constructed for circRNA and miRNA sequencing. The rate of reads mapped to the reference genome of *A. albopictus* was 42.17% to 67.01% for circRNA and 72.38% to 96.25% for miRNA (Additional file 2: Tables S1, S2). The Q30 of all samples was above 90.98%. Raw circRNA and miRNA data were deposited in the NCBI Sequence Read Archive (SRA) database with accession numbers PRJNA863740 (miRNA) and PRJNA757239 (circRNA).

After normalization and filtration, a total of 1434 circRNAs and 208 miRNAs were identified (Additional file 2: Table S3). All 1434 circRNAs are novel circRNAs, and there were 1262 exon_circRNAs, 138 intergenic_region_circRNAs, and 34 intron_circRNAs according to their localization in the genome (Additional file 2: Table S3). A total of 125 known miRNAs and 83 novel miRNAs were identified. Venn analysis suggested that 148 circRNAs were detected at all developmental time points, and 87, 30, 89, 86, 197, and 40 circRNAs were unique to E, L1, L2, P, M, and F, respectively (Additional file 1: Fig. S1a). Analysis of the miRNAs showed that only 33 miRNAs were present at all developmental time points, and most of the miRNAs were expressed at three or more developmental time points (Additional file 1: Fig. S1b).

**Fig. 1:**
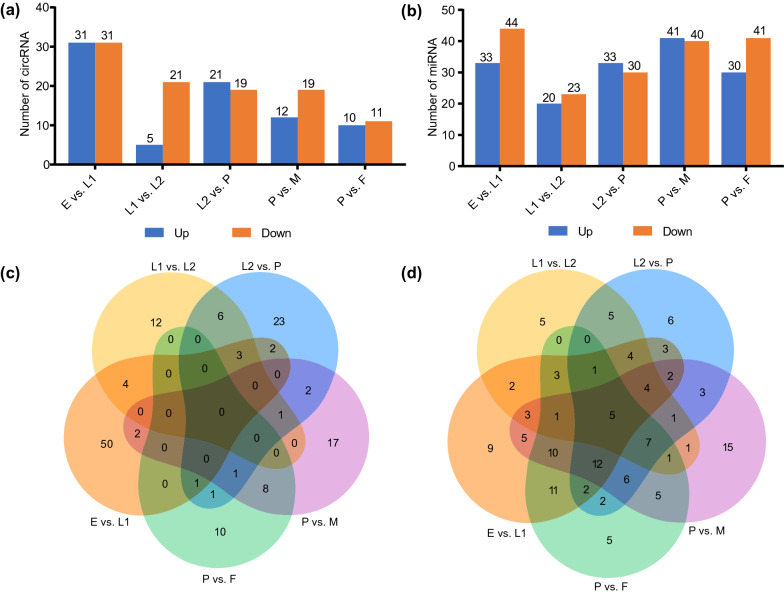
Expression profiles of circRNAs and miRNAs. Histogram showing DE circRNAs (**a**) and DE miRNAs (**b**) among successive developmental time points; Venn diagram displaying the number of overlapping DE circRNAs (**c**) and DE miRNAs (**d**) between different groups. *E* egg, *L1* early larvae, *L2* late larvae, *P* pupae, *F* female, *M* male

According to the successive developmental time points of *A. albopictus*, five comparison groups were assigned: egg versus early larvae (E vs. L1), early larvae versus late larvae (L1 vs. L2), late larvae versus pupae (L2 vs. P), pupae versus female (P vs. F), and pupae versus male (P vs. M). In total, 143 DE circRNAs were detected. The same number (31 circRNAs) of DE circRNAs was up- and downregulated in the E vs. L1 comparison group; more DE circRNAs were downregulated in the L1 vs. L2, P vs. M, and P vs. F comparison groups, while more upregulated DE circRNAs were detected in the L2 vs. P comparison group (Fig. 1a DE circRNA). There were 139 DE miRNAs, and many more DE miRNAs were downregulated in the E vs. L1, L1 vs. L2, and P vs. F comparison groups; the number of upregulated DE miRNAs exceeded the number of downregulated DE miRNAs in the L2 vs. P and P vs. M comparison groups (Fig. 1b DE miRNA). There were 50, 12, 23, 17, and 10 DE circRNAs specifically expressed in the E vs. L1, L1 vs. L2, L2 vs. P, P vs. M, and P vs. F comparison groups, respectively (Fig. 1c Venn DE circRNA). Five DE miRNAs (aal-miR-305-5p, aal-miR-279, aal-miR-957, aal-miR-282-5p, and aal-miR-263a-5p) were found expressed at all six developmental time points. There were 9, 5, 6, 15, and 5 DE miRNAs unique to the E vs. L1, L1 vs. L2, L2 vs. P, P vs. M, and P vs. F comparison groups, respectively (Fig. 1d Venn DE miRNA).

### ceRNA network construction

The circRNA-associated ceRNA networks of the E vs. L1, L1 vs. L2, L2 vs. P, P vs. M, and P vs. F comparison groups included 50, 16, 28, 18, and 16 DE circRNAs, respectively (Additional file 2: Table S4). The highest number of nodes (3557, including 50 DE circRNAs, 3442 DE mRNAs, and 65 DE miRNAs) and edges (13,573) was detected in the ceRNA network of the E vs. L1 group; the lowest number of nodes (431, including 16 DE lncRNAs, 390 DE mRNAs and 25 DE miRNAs) and edges (924) were found in the ceRNA network of the L1 vs. L2 group (Additional file 2: Table S4). In addition, according to the connective degree of each node in the networks, all of the top 10 hub genes in each group were miRNAs, and several of them were shared among groups, such as aae-miR-285, aae-miR-305-5p, and aae-miR-34-5p (Additional file 2: Table S5).

In the lncRNA-associated ceRNA networks, most of the nodes and edges were also found in the ceRNA network of the E vs. L1 comparison group, which consisted of 1604 DE lncRNAs, 3692 DE mRNAs and 77 DE miRNAs (Additional file 2: Table S6). Similar to that of the circRNA-associated ceRNA network, only 816 nodes (308 DE lncRNAs, 465 DE mRNAs, and 43 DE miRNAs) and 2508 edges were included in the lncRNA-associated ceRNA network of the L1 vs. L2 comparison group (Additional file 2: Table S7). The top 10 hub genes in all lncRNA-associated ceRNA networks were miRNAs, and aae-miR-305-5p was detected in all groups; aae-miR-34-5p, aae-miR-285, aae-miR-92a-3p, and aae-miR-92b-3p were found in the L1 vs. L2, L2 vs. P, P vs. M, and P vs. F comparison groups (Additional file 2: Table S7).

### Functional enrichment of the ceRNA networks

To assess the putative regulatory roles of the circRNAs and miRNAs at different developmental time points, functional enrichment analysis was performed for all DE mRNAs in the circRNA- and lncRNA-associated ceRNA networks.

Overrepresented GO terms and KEGG pathways reflected stage-specific key biological processes during the development of *A. albopictus*. The top 20 enriched GO terms and KEGG pathways are listed in Additional file 3: Tables S8–S11. Notably, the top 20 enriched KEGG pathways were primarily metabolism-related, including “alanine, aspartate and glutamate metabolism (map00250),” “nitrogen metabolism (map00910),” “carbon metabolism (map01200),” and “biosynthesis of unsaturated fatty acids (map01040),” in both the circRNA- and lncRNA-associated ceRNA networks. Highly enriched GO terms including “structural constituent of cuticle (GO: 0042302),” “endopeptidase activity (GO: 0004175),” “proteolysis (GO: 0006508),” “serine hydrolase activity (GO: 0017171),” and “hydrolase activity (GO: 0016787)” were found in several circRNA- and lncRNA-associated ceRNA networks.

GO term annotation analysis showed that circRNA-associated ceRNA networks were significantly enriched in “structural constituent of cuticle,” which was the most enriched GO term in all four comparison groups (E vs. L1, L1 vs. L2, P vs. M, and P vs. F); “peptidase activity, acting on L-amino acid peptides (GO: 0070011),” was the most highly enriched GO term in the L2 vs. P comparison group. KEGG pathway enrichment analysis showed that “nitrogen metabolism” was the most enriched pathway in the E vs. L1, L2 vs. P, P vs. M, and P vs. F comparison groups. In the L1 vs. L2 comparison group, “arginine and proline metabolism (map00330)” was the most enriched pathway. Functional annotation of lncRNA-associated ceRNA networks showed a similar pattern to that of the circRNA-associated ceRNA networks. “Structural constituent of cuticle” was the most enriched GO term in the E vs. L1, L1 vs. L2, P vs. M, and P vs. F comparison groups. Meanwhile, “serine-type peptidase activity (GO: 0008236)” and “serine hydrolase activity” were the top two significantly enriched GO terms in the L2 vs. P comparison group. “Nitrogen metabolism (map00910)” was the most enriched pathway in the E vs. L1, P vs. M, and P vs. F comparison groups. “Arginine and proline metabolism (map00330)” and “carbon metabolism” were the highly enriched pathways in the L1 vs. L2 and L2 vs. P comparison groups, respectively.

**Fig. 2:**
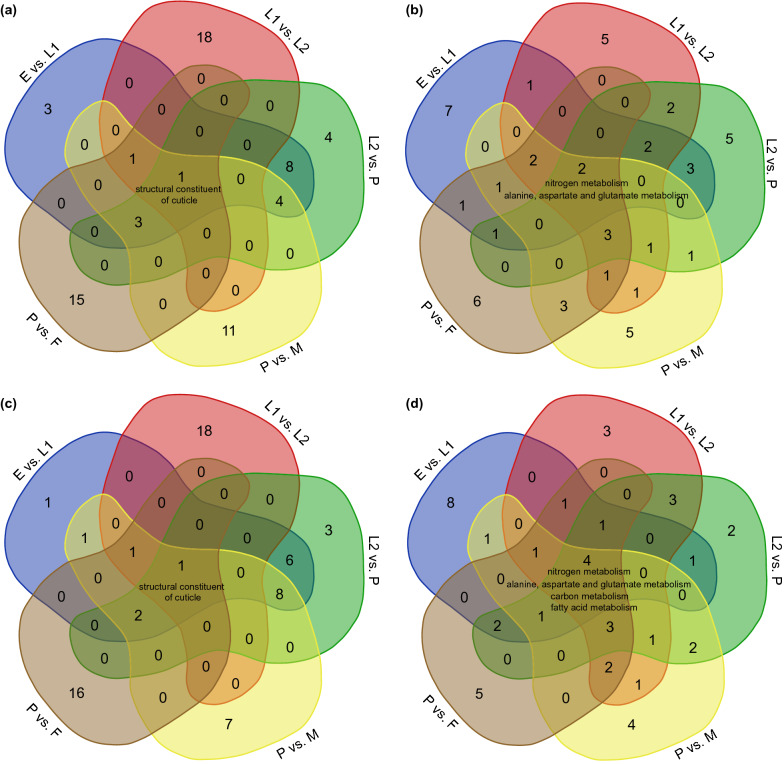
Venn diagram showing the enriched top 10 GO terms and KEGG pathways in circRNA- and lncRNA-associated ceRNA networks of different comparison groups. Co-expressed GO terms of circRNA-associated ceRNA networks (**a**); co-expressed KEGG pathways of circRNA-associated ceRNA networks (**b**); co-expressed GO terms of lncRNA-associated ceRNA networks (**c**); co-expressed KEGG pathways of lncRNA-associated ceRNA networks (**d**). *E* egg, *L1* early larvae, *L2* late larvae, *P* pupae, *F* female, *M* male

Stage-specific and shared functional annotations of DE circRNAs and DE miRNAs were obtained by conducting Venn analysis. Based on functional annotation of all circRNA- and lncRNA-associated ceRNA networks, the “structural constituent of cuticle” GO term was enriched in all five comparison groups (Fig. 2a, c). “Nitrogen metabolism” and “alanine, aspartate and glutamate metabolism” were KEGG pathways highly enriched in all comparison groups (Fig. 2b, d). Ultimately, the overlapping GO terms (structural constituent of cuticle) and KEGG pathways (nitrogen metabolism, alanine, aspartate, and glutamate metabolism) that were highly enriched in the circRNA–miRNA–mRNA networks and lncRNA–miRNA–mRNA networks were used for further investigation.

### Identification of hub genes in the co-expressed key functions

Based on the results of functional enrichment analysis, ncRNAs of all developmental time points were integrated to construct ceRNA networks that participated in the regulation of “structural constituent of cuticle,” “nitrogen metabolism,” and “alanine, aspartate and glutamate metabolism.” Meanwhile, hub genes of each network were obtained according to their connective degree in the networks.

**Fig. 3:**
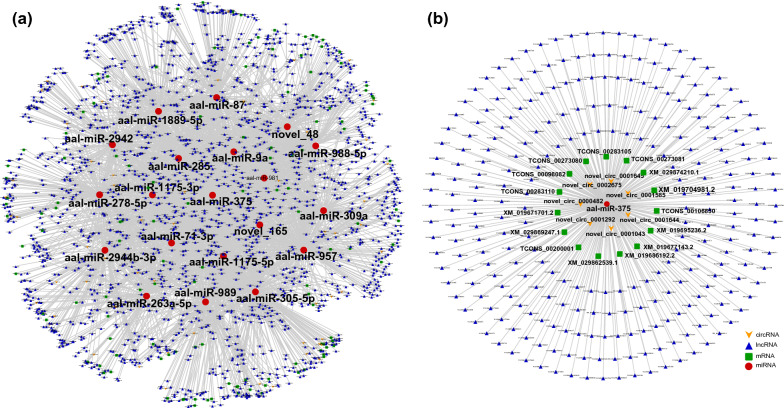
Constructed ceRNA networks based on ncRNAs involved in the “structural constituent of cuticle” (**a**) and subnetwork of miR-375 (**b**). Each node represents a ncRNA, each edge denotes a target relationship between ncRNAs. The yellow arrow represents circRNA, the blue triangle represents lncRNA, the green square represents mRNA, and the red dot represents miRNA

**Fig. 4:**
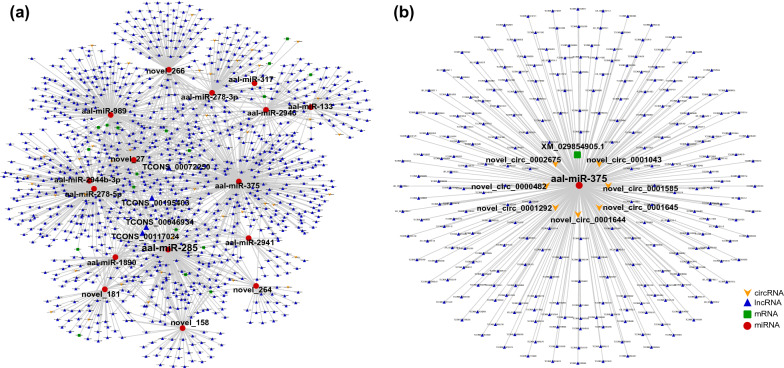
Constructed ceRNA networks based on ncRNAs involved in “nitrogen metabolism” (**a**) and subnetwork of miR-375 (**b**). Each node represents a ncRNA, each edge denotes a target relationship between ncRNAs. The yellow arrow represents circRNA, the blue triangle represents lncRNA, the green square represents mRNA, and the red dot represents miRNA

**Fig. 5:**
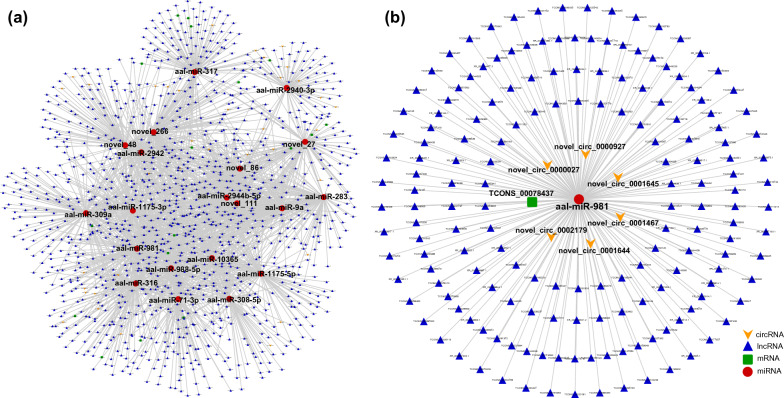
Constructed ceRNA networks based on ncRNAs involved in “alanine, aspartate and glutamate metabolism” (**a**) and subnetwork of miR-981 (**b**). Each node represents a ncRNA, each edge denotes a target relationship between ncRNAs. The yellow arrow represents circRNA, the blue triangle represents lncRNA, the green square represents mRNA, and the red dot represents miRNA

Altogether, 2753 nodes (94 circRNAs, 2344 lncRNAs, 116 miRNAs, 199 mRNAs) and 10,019 edges were involved in the ceRNA networks of the “structural constituent of cuticle” (Fig. 3a; Additional file 3: Table S12). All of the top 20 hub genes were miRNAs, and aae-miR-375 had the highest connective degree (Additional file 4: Table S13). In the subnetwork of aae-miR-375, there were 290 nodes (7 circRNAs, 268 lncRNAs, and 15 mRNAs) (Fig. 3b). In the ceRNA networks that participate in the regulation of “nitrogen metabolism,” 1121 nodes (41 circRNAs, 1044 lncRNAs, 17 miRNAs, 19 mRNAs) and 1509 edges were included. Among the top 20 hub genes, there were 16 miRNAs and four lncRNAs (Fig. 4a; Additional file 4: Table S12). Notably, the connective degree of aae-miR-375 is also at the top of the lists (Additional file 4: Table S13). In this ceRNA network, the subnetwork of aae-miR-375 consisted of 178 nodes (6 circRNAs, 171 lncRNAs, 116 miRNAs, 1 mRNA) (Fig. 4b). The ceRNA networks involved in “alanine, aspartate and glutamate metabolism” consisted of 1477 nodes (44 circRNAs, 1377 lncRNAs, 34 miRNAs, and 22 mRNAs) and 2730 edges (Fig. 5a; Additional file 4: Table S12). The connective degree of aae-miR-981 was ranked first among all nodes (Additional file 4: Table S13). Altogether, 277 nodes (7 circRNAs, 268 lncRNAs, and 1 mRNA) were included, and 276 edges constituted the subnetwork of aae-miR-981 (Fig. 5b).

### qRT-PCR confirmation of differentially expressed miRNAs

**Fig. 6:**
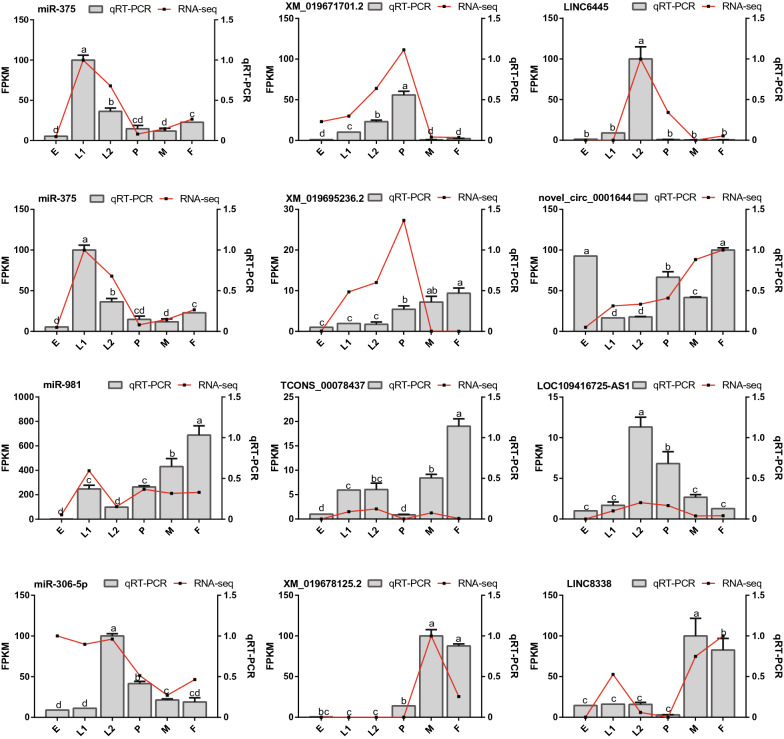
Comparison of expression profiles of ncRNAs involved in four ceRNA networks RNA-seq and qRT-PCR analyses. The *x*-axis represents developmental stages; the red lines represents the results of the RNA-seq, and the columns represent the results of the qRT-PCR. *E* egg, *L1* early larvae, *L2* late larvae, *P* pupae, *F* female, *M* male

To validate the reliability of the RNA-seq data, four ceRNA networks (LINC6445-miR-375-XM019671701.2, novel circ0001644-miR-375-XM019695236.2, LOC109416725-AS1-miR 981-TCONS00078437, and LINC8338-miR-306-5p-XM019678125.2) were randomly selected for further qRT-PCR analyses. The primers used in this study are listed in Table S14. The changes in the expression levels of miRNAs, lncRNAs, circRNAs, and mRNAs included in these four ceRNA networks showed similar trends as the RNA-seq data (Fig. 6), confirming the reliability of the RNA-seq data and guaranteeing the accuracy of the related analysis.

## Discussion

As one of the most important classes of post-transcriptional regulatory factors, several studies have addressed the identification, characterization, and function of miRNAs and confirmed their roles in the growth, development, and immunity of insects [16]. Nevertheless, the expression profiles, functions, and mechanisms of ncRNAs in *A. albopictus* remain largely unknown. In the current study, circRNA, lncRNA, and miRNA data were combined to obtain a comprehensive profile of ncRNAs involved in the development of *A. albopictus*.

Expression pattern analysis demonstrated that most of the circRNAs and miRNAs were differentially expressed at five developmental time points in *A. albopictus*. The dynamic changes in the DE circRNAs and DE miRNAs were significantly different between the comparison groups, with the most DE circRNAs and DE miRNAs all found in the E vs. L1 comparison group (Fig. 1a, b). A similar result was also detected for the lncRNAs [26]. Consistent with the dynamic pattern of DE ncRNAs, the highest number of nodes in both circRNA- and lncRNA-associated ceRNA networks was in the E vs. L1 group (Additional file 2: Table S4, S6). These results suggested complex regulatory relationships among ncRNAs during the transition from eggs to early larvae. As a holometabolous insect, the biological cycle of *A. albopictus* involves egg, larval, pupal, and adult stages. Embryogenesis of the mosquito is a complex process involving rapid cell proliferation and organogenesis [46]. Therefore, it makes sense that a relatively high proportion of differentially expressed genes (DEGs) and more complex ceRNA networks were identified in the E vs. L1 comparison group. Meanwhile, the fewest ceRNA network nodes were found in the L1 vs. L2 comparison group, suggesting simpler regulatory relationships within the same developmental stage.

Although the exact functions of the majority of ncRNAs remain unclear, directly linked genes in ceRNA networks usually have similar functions and may be involved in the same biological process. Correspondingly, in a specific network, the higher the connection degree of a node, the more important this gene might be. Functional enrichment analyses revealed that most of the circRNA- and lncRNA-associated ceRNA networks were metabolism-related. As is well known, during the metamorphic development of insects, either the larva molts from one instar to the next or the final larval instar undergoes a metamorphic molt to pupa to adult, combined with extensive remodeling of organs and tissues, and in some cases the organism is completely rebuilt [47]; thus, metabolism-related biological processes are necessary to provide materials such as energy and nutrients to support the metamorphic development of *A. albopictus*.

All insects produce exoskeletons, which are essential in all developmental stages to protect the insect from mechanical injury and predation. Due to growth and development, the old exoskeleton becomes a physical constraint, and the insect synthesizes a new cuticle and partially digests and sheds the cuticle from the previous developmental stage during each molt [48–51]. It has been observed that the growth of insects is highly dependent on their capability for remodeling of the exoskeleton. Chitin is a major component of the cuticle that forms the exoskeleton and the internal chitinous structures such as the peritrophic matrix (PM) of the midgut [50, 52, 53]. The periodic synthesis and degradation of chitin accompanying each molt explains the highly enriched “structural constituent of cuticle” GO terms detected in almost all developmental stages of *A. albopictus*.

Chitin is the most abundant nitrogen-containing biopolymer on Earth [54]. As a nitrogenous polysaccharide, chitin is a linear homopolymer composed of β-1,4-linked subunits of *N*-acetylglucosamine [55]. Therefore, the predominant “nitrogen metabolism” pathways in both circRNA- and lncRNA-associated ceRNA networks fulfill the requirements for chitin synthesis during the life-cycle of *A. albopictus*. Additionally, molting is a high-energy-demanding event. The energy is needed for the disruption of and emergence from the old cuticle, and a new cuticle has to be built toward the end of metamorphosis (larva to larva, larva to pupa, pupa to adult) [56]. As one of the most important energy metabolism pathways, “nitrogen metabolism” might also participate in the energy supply during the metamorphic development of *A. albopictus*.

Functional annotation of both circRNA- and lncRNA-associated ceRNA networks revealed active amino acid metabolism, including the “alanine, aspartate and glutamate metabolism” KEGG pathway, which was highly enriched in all developmental stages of *A. albopictus*. Amino acids are the main building blocks of proteins [57]. It is speculated that these amino acids might participate in the formation of chitin-binding proteins, which is another important component of the cuticle in addition to chitin. Chitin-binding proteins together with lipids, carbohydrates, and other minor components depend largely on the mechanical properties of the cuticle [58, 59]. Similar results were also found in the molting of *Helicoverpa armigera* at the proteomic level. Upregulated proteins in larval and metamorphic molting of *H. armigera* were found to be enriched in the amino sugar metabolism pathway, lipid transport process, and protein catabolism, which are related to the metabolism of chitin [60].

Molting of insects involves both the synthesis of chitin in the new procuticle and the degradation of chitin (and protein) in the innermost parts of the old cuticle [50]. The digestion of chitin in the old cuticle requires the participation of several enzymes, including an assortment of proteolytic enzymes [61, 62]. This explains the highly enriched GO terms “endopeptidase activity (GO: 0004175),” “proteolysis (GO: 0006508),” “serine hydrolase activity (GO: 0017171),” and “hydrolase activity (GO: 0016787)” found both in circRNA- and lncRNA-associated ceRNA networks.

As shown above, ncRNAs play vital roles in the chitin metabolism of *A. albopictus*, especially “structural constituent of cuticle,” “nitrogen metabolism,” and “alanine, aspartate, and glutamate metabolism,” which are crucial for chitin synthesis and were highly enriched in all developmental stages. Therefore, key ncRNAs involved in these three biological processes would be the optimal choice for altering the formation of the cuticle. However, ncRNAs may regulate multiple targets in different ceRNA networks, and vice versa: one gene can be regulated by multiple ncRNAs [19]. Because some ncRNAs exhibited stage specificity, ncRNAs at all developmental time points involved in the above three chitin metabolism-related biological processes were integrated to provide a comprehensive understanding of the interactions between the ncRNAs (Figs. 3a, 4a, 5a). In both the “structural constituent of cuticle” and “nitrogen metabolism” regulatory networks, aae-miR-375 was found to have the highest connectivity degree, suggesting a crucial role of aae-miR-375 in chitin metabolism. In addition, aae-miR-981 was the node with the highest connectivity in the regulatory network of “alanine, aspartate and glutamate metabolism.” Therefore, the miRNA–target gene interaction network of aae-miR-375 (Figs. 3b, 4b) and aae-miR-981 (Fig. 5b) was constructed to obtain more detailed regulatory relationships centered on this gene. The subnetwork of aae-miR-375 and aae-miR-981 provided more information for exploring the extensive regulatory roles of these two hub genes. Combined with the top 10 hub genes in each ceRNA network of different comparison groups (Additional file 2: Table S5), this study provided many vital targets for disturbing the molting process of *A. albopictus* in future work. Hub genes that are conserved in all developmental stages, such as aae-miR-375 and aae-miR-981, allow intervention in normal growth and development of *A. albopictus* at any stage, while hub genes specific to certain stages can be used as stage-specific tuners to disrupt the normal molting process and achieve the goal of controlling mosquitoes and mosquito-borne diseases.

Taken together, a comprehensive catalog of ncRNAs across five developmental stages of *A. albopictus* was produced in the present study. ceRNA networks revealed interplay among circRNAs, lncRNAs, and miRNAs, improving our knowledge of the post-transcriptional regulations involved in the development of *A. albopictus*. Functional classifications of most typical ceRNA networks were linked to cuticle formation and chitin metabolism. As chitin is an indispensable structure for insects and is one of the structural components essential for individual growth and development, research on exploiting targets to disrupt the biosynthesis and regulatory pathways of chitin would be helpful for developing new insect control technologies. Previous studies have attempted to inhibit chitin synthesis in insects mainly by interrupting chitin synthase using RNA interference (RNAi) technology [63–66]. Some studies have shown that miRNAs are involved in chitin biosynthesis and regulate gene expression during the molting of insects [67–70]. The present study revealed roles of ncRNAs in chitin metabolism, providing more molecular targets for regulating chitin-related biological processes. To be specific, hub genes of each developmental stage were provided; in addition to miRNAs, some lncRNAs and circRNAs were also found as hub genes, which might be used as new targets to manipulate chitin metabolism. More importantly, aae-miR-981 and aae-miR-375 were shown to be crucial hub genes involved in three important chitin metabolism pathways that are highly enriched in all developmental stages. The results generated in this study can be a starting point for dissecting the mechanisms of ncRNA functions in *A. albopictus*, chitin metabolism-related hub genes and the regulatory relationship between ncRNAs, which may act as new target sites to shed light on possible strategies for mosquito control. If chitin metabolism is dysregulated, such as failure of degradation of old cuticles or synthesis of new ones, the growth and development of the insect will be blocked.

## Conclusion

This study provides comprehensive insight into ncRNAs across different developmental stages of *A. albopictus*. DE circRNAs, DE miRNAs, and circRNA- and lncRNA-associated ceRNA networks for five comparison groups were obtained. Functional annotation suggested that both circRNA- and lncRNA-associated ceRNA networks played crucial roles in chitin metabolism. “Structural constituent of cuticle,” “nitrogen metabolism,” and “alanine, aspartate and glutamate metabolism,” which are important chitin metabolism-related biological processes, were highly enriched in most developmental stages of *A. albopictus*. Hub genes of each ceRNA network were screened out, which can be inferred as promising candidate molecular target sites to interrupt chitin metabolism, and used for developing safe, effective, and sustainable mosquito control methods.

## Supplementary Information

**Additional file 1: Figure S1.** Venn diagram showing the number of circRNA (a) and miRNA (b) in different developmental stages. E, egg; L1, early larvae; L2, late larvae; P, pupae; F, female; M, male.

**Additional file 2: Table S1**. Overview of the data for RNA sequencing. **Table S2**. Overview of the data for small RNA sequencing. **Table S3**. Identified miRNAs and circRNA. **Table S4**. Statistics of nodes and edges in ceRNA networks. **Table S5**. Top 10 hub genes in the ceRNA network of different groups. **Table S6**. Statistics of nodes and edges in lncRNA-associated ceRNA networks. **Table S7**. Top 10 hub genes in the lncRNA-associated ceRNA network of different groups.

**Additional file 3: Table S8**. Top 20 GO terms in circRNA-associated ceRNA networks of different comparison groups. **Table S9**. Top 20 KEGG pathways in circRNA-associated ceRNA networks of different comparison groups. **Table S10**. Top 20 GO terms in lncRNA-associated ceRNA networks of different comparison groups. **Table S11**. Top 20 KEGG pathways in lncRNA-associated ceRNA networks of different comparison groups.

**Additional file 4: Table S12**. Information of ceRNA networks of three co-expressed biological processes in different developmental stages. **Table S13**. Top 20 nodes with the highest connectivity degree in three co-expressed biological processes in different developmental stages. **Table S14**. Primers used for qRT-PCR in this study.

## Data Availability

The authors declare that all the data related to this study are available within the paper or can be obtained from the authors upon reasonable request.
