# Supplementary figures and images for: Whole-transcriptome profiling across different developmental stages of Aedes albopictus (Diptera: Culicidae) provides insights into chitin-related non-coding RNA and competing endogenous RNA networks
Source: Parasit Vectors. 2023 Jan 26;16:33. doi: 10.1186/s13071-022-05648-2 (PMC9878986; doi:10.1186/s13071-022-05648-2)

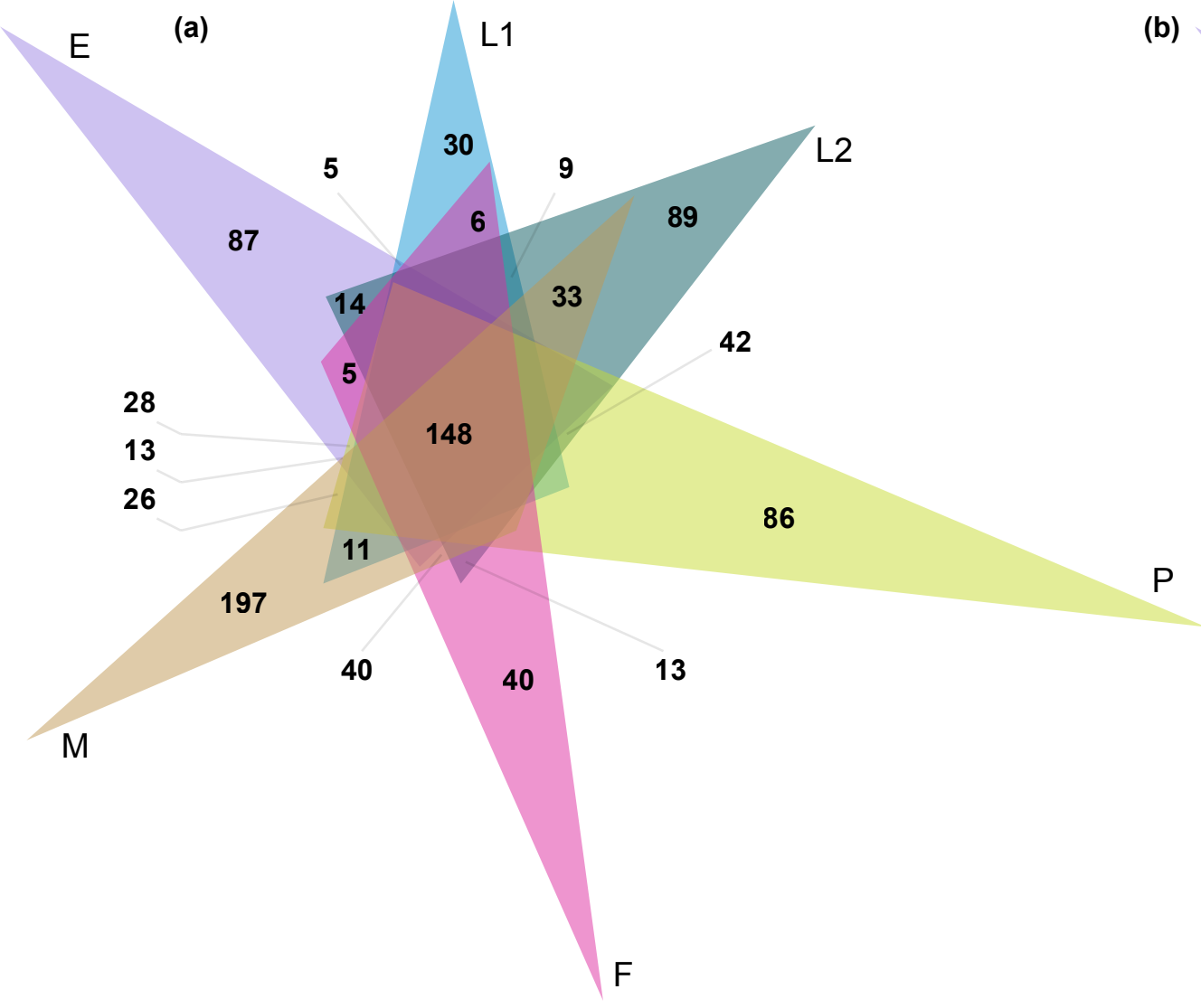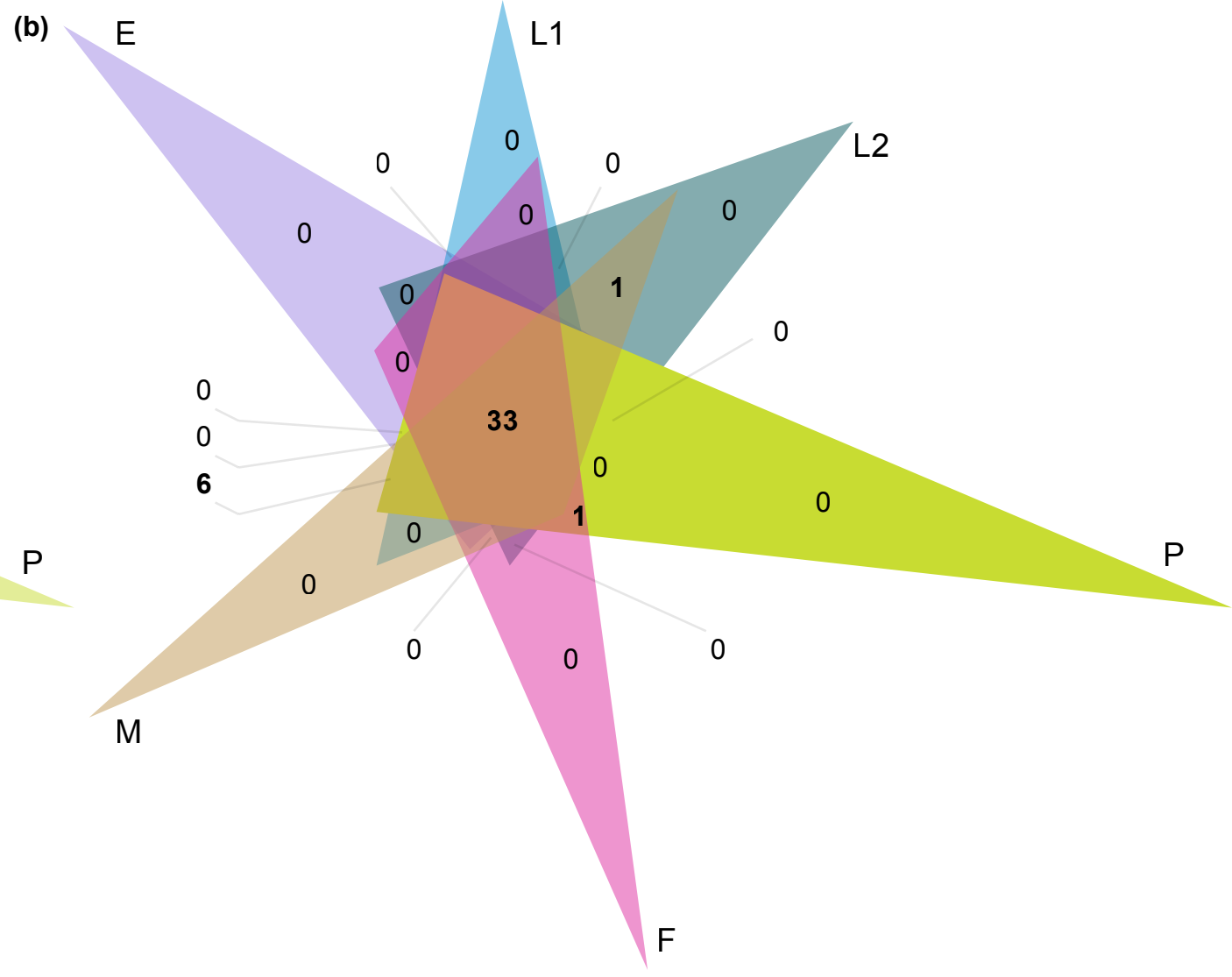

Supplement: Supplementary file 1 — Additional file 1: Figure S1. Venn diagram showing the number of circRNA (a) and miRNA (b) in different developmental stages. E, egg; L1, early larvae; L2, late larvae; P, pupae; F, female; M, male. [file 13071_2022_5648_MOESM1_ESM.pdf]
